# Association between prevalence of obstructive lung disease and obesity: results from The Vermont Diabetes Information System

**DOI:** 10.1186/s40733-021-00073-1

**Published:** 2021-05-28

**Authors:** Maria E. Ramos-Nino, Charles D. MacLean, Benjamin Littenberg

**Affiliations:** 1 Department of Microbiology, Immunology, and Pharmacology, St. George’s University, West Indies, Grenada; 2 Department of Pathology and Laboratory Medicine, University of Vermont 05401, Burlington, VT USA; 3 Department of Medicine, University of Vermont, Burlington, VT 05401 USA

**Keywords:** Obstructive lung disease, Asthma, COPD, Obesity, Diabetes

## Abstract

**Background:**

The association of obesity with the development of obstructive lung disease, namely asthma and/or chronic obstructive pulmonary disease, has been found to be significant in general population studies, and weight loss in the obese has proven beneficial in disease control. Obese patients seem to present with a specific obstructive lung disease phenotype including a reduced response to corticosteroids. Obesity is increasingly recognized as an important factor to document in obstructive lung disease patients and a critical comorbidity to report in diabetic patients, as it may influence disease management. This report presents data that contributes to establishing the relationship between obstructive lung disease in a diabetic cohort, a population highly susceptible to obesity.

**Methods:**

A total of 1003 subjects in community practice settings were interviewed at home at the time of enrolment into the Vermont Diabetes Information System, a clinical decision support program. Patients self-reported their personal and clinical characteristics, including any history of obstructive lung disease. Laboratory data were obtained directly from the clinical laboratory, and current medications were obtained by direct observation of medication containers. We performed a cross-sectional analysis of the interviewed subjects to assess a possible association between obstructive lung disease history and obesity.

**Results:**

In a multivariate logistic regression model, a history of obstructive lung disease was significantly associated with obesity (body mass index ≥30) even after correcting for potential confounders including gender, low income (<$30,000/year), number of comorbidities, number of prescription medications, cigarette smoking, and alcohol problems (adjusted odds ratio (OR) = 1.58, *P* = 0.03, 95% confidence interval (CI) = 1.05, 2.37). This association was particularly strong and significant among female patients (OR = 2.18, *P* = < 0.01, CI = 1.27, 3.72) but not in male patients (OR = 0.97, *P* = 0.93, CI = 0.51, 1.83).

**Conclusion:**

These data suggest an association between obesity and obstructive lung disease prevalence in patients with diabetes, with women exhibiting a stronger association. Future studies are needed to identify the mechanism by which women disproportionately develop obstructive lung disease in this population.

## Background

Obesity, defined by the WHO as a body mass index (BMI) > 30 kg/m^2^, is a worldwide epidemic [1]. In 2017–2018, the age-adjusted prevalence of obesity in US adults was 42.4%, with no significant differences between men and women or across age groups [2]. The age-adjusted prevalence of severe obesity (BMI ≥ 40 kg/m^2^) in adults was 9.2% and was higher in women than in men [2]. Obesity can lead to increased health risks associated with high morbidity and premature mortality [3–7]. Obesity-related mortality is second only to tobacco-related mortality as the leading cause of preventable death in the United States [8].

Obesity is a well-known risk factor for cardiovascular disease, type 2 diabetes, and some forms of cancer [9]; however, it is increasingly apparent that obesity is also a risk factor for the development of certain respiratory diseases [10].

Chronic lower respiratory disease, which includes emphysema, chronic bronchitis, asthma, pulmonary hypertension, and occupational lung diseases comprise the fourth-leading cause of death in the United States [11]. Obstructive lung disease, a component of chronic lower respiratory disease, includes asthma and chronic obstructive pulmonary disease (emphysema and chronic bronchitis) [12]. Asthma is a major cause of morbidity in the United States, affecting 7.7% of the US population, with a female preponderance (9.1% of women vs. 6.1% of men (CDC, 2018). The annual cost of asthma is more than $80 billion in medical expenses, missed work and school days, and deaths [13]. Much of this cost is related to the burden of acute exacerbations [14].

Several studies have described an association between BMI and asthma in adults [15–17], particularly in women [18–21]. For example, among 19,126 Dutch adults, obese women were nearly twice as likely to have asthma as compared with those of normal body mass [22].

Prospective studies showing an association between obesity and asthma have also been reported. One such study included 85,911 women subjects that were followed for a period of 4 years. The authors reported a strong, dose–response relationship between BMI and the relative risk of new-onset asthma [23]. Other prospective studies have also reported that obesity precedes the development of asthma [16, 24]. As with the cross-sectional studies, many of these prospective studies showed a stronger effect in women than men.

Obese patients with asthma are 2.8 times more likely to have poor asthma-specific quality of life, 2.7 times more likely to have poor asthma control, and 5 times more likely to be hospitalized for asthma than the non-obese (BMI under 25 kg/m^2^) even after adjusting for confounding variables [25]. Furthermore, weight loss is associated with an improvement in some measures of asthma status [24]. Although these studies are not definitive, the consistency of the results provides some evidence to support a causal relationship between obesity and asthma.

There are also studies that report an association between obesity and Chronic Obstructive Pulmonary Disease (COPD), which includes chronic bronchitis and emphysema. However, in overweight patients, a paradox appears to exist. Obesity does not worsen the respiratory function symptoms of COPD [26] and seems to have a protective effect in relation to mortality, but results are different in morbid obesity (BMI ≥40 kg/m^2^) [27]. One study reported a significant increase in deaths due to respiratory disease in subjects with a BMI ≥40 kg/m^2^ (hazard ratio = 5.78, 95% confidence interval (CI) = 1.09, 30.61). Diabetes is a common comorbidity of COPD [27] and is associated with impaired lung function [28]. Nonetheless, the association between COPD and diabetes has not been fully established epidemiologically or clinically.

In this cross-sectional study, we sought to describe the association between obesity and obstructive lung disease (asthma, emphysema, and bronchitis) in a cohort of primary care patients with diabetes.

## Methods

This study is part of a larger project, the Vermont Diabetes Information System (VDIS), a study of 7412 adults with diabetes in primary care practices [29]. The subjects comprised all diabetic adults in 64 practices in Vermont and adjacent New Hampshire and New York. A field survey was completed in 2003–2005 with a random subsample of subjects. Of 1007 surveyed subjects, four were dropped due to incomplete information, leaving a final sample of 1003.

Subjects completed a questionnaire at home and then were visited by a trained research assistant who reviewed the questionnaire responses, assisted the subject with any missing or unacceptable responses, reviewed the subject’s medications, and measured their height and weight using a portable stadiometer and scale. Race, education, income, marital status, functional status, smoking, alcohol consumption, and comorbid conditions were obtained by questionnaire. The primary outcome variable, presence of obstructive lung disease, was the patient’s response to the question “Do you have asthma, emphysema, or chronic bronchitis?” The primary predictor variable was obesity defined as BMI > 30 kg/m^2^.

To determine comorbidity, we used a modification of the Self-Administered Comorbidity Questionnaire [30] in which we asked each patient to indicate whether they had had the following conditions: heart attack, heart failure, peripheral arterial disease, stroke, dementia, rheumatic disease, peptic ulcer, cirrhosis, paralysis, renal insufficiency, diabetic vascular complications, AIDS/HIV, and depression. All patients also had diabetes, which was not included in the comorbidity count. Most laboratory data were obtained from the patients’ local clinical laboratories, which all used the same Diabetes Control and Complications Trial/Epidemiology of Diabetes Interventions and Complications high performance, liquid chromatography (HPLC) method for the determination of glycosylated hemoglobin (A1C). Less than 1% of A1C tests were performed using the Bayer DCA 2000 immunoassay point of care instrument, which compares favorably with the HPLC method [31].

The research protocol was approved by the Committee on Human Research of the University of Vermont. The interviewed subjects provided written informed consent. The full study protocol and variables and the medication profiles of the subjects have been previously reported [29, 32].

### Statistical approach

We used logistic regression to assess the univariate relationship of obstructive lung disease as the outcome variable with obesity (BMI ≥30 kg/m^2^) as the predictor. We then adjusted for possible confounding by social and clinical factors. Potential confounders tested were gender (male/female), age (years), race (White/other), glycosylated hemoglobin level (A1C; %), insulin use (yes/no), self-reported history of alcohol problems (yes/no), cigarette smoking (yes/no), current estrogen use (yes/no), low annual income (<$30,000 per year), duration of diabetes in years, number of comorbidities (excluding lung disease and diabetes), and number of prescription medications. To reduce the number of variables in the final model, we excluded potential confounders that were associated with the outcome in univariate analyses with *P* > 0.15. Such a weak association implies that the variable is unlikely to be a confounder. Because the association between diabetes and obesity has a strong gender component, we also performed separate multivariate analyses for men and women. We used Stata/SE v.14 (StataCorp, College Station, TX, USA) for all analyses.

## Results

The study population was representative of adults with diabetes in primary care practices in northern New England, USA. One in five reported a history of lung disease, and two-thirds were obese (Table 1).

**Table 1:** Baseline characteristics of 1003 adults with diabetes

Characteristic	N (%) or Mean (SD)
History of obstructive lung disease	203 (20.2%)
Body mass	
BMI, kg/m^2^	33.8 (7.4)
Normal weight (BMI 18.5–24.9 kg/m^2^)	87 (8.8%)
Overweight (BMI 25–29.9 kg/m^2^)	223 (22.5%)
Obese (BMI > 30 kg/m^2^)	666 (67.3%)
Gender	
Men	457 (45.6%)
Women	546 (54.4%)
Age, years	64.8 (12.0)
White race	973 (97.3%)
Glycosylated hemoglobin A1C, %	7.13 (1.3)
Insulin use	185 (18.4%)
Alcohol use	274 (27.4%)
Alcohol problem	78 (7.9%)
Cigarette smoking	170 (16.9%)
Estrogen use	42 (4.19%)
Median income ($/year)	15,000–29,999
Low annual income (<$30,000)	548 (59.1%)
Duration of diabetes, years	10.2 (10.3)
Number of comorbidities	1.7 (1.7)
Number of prescription medications	6.7 (3.8)

*SD* Standard deviation, *N* Number of subjects with the characteristic.

Table 2 presents univariate associations between obstructive lung disease and the other study variables that had the potential of being significantly associated with the presence of obstructive lung disease.

**Table 2:** Univariate associations between history of obstructive lung disease and other patient characteristics

Characteristic	Obstructive lung disease patients	Non-obstructive lung disease patients	Unadjusted Odds Ratio	***P***
	% or mean (SD)	% or mean (SD)		
Number of subjects	203	800		
Male, %	33.5%	48.6%	0.53	< 0.01
Age, years	64.3 (11.4)	64.9 (12.1)	1.00	0.54
White race	96.1%	97.6%	0.60	0.23
A1C, mg %	7.2 (1.3)	7.1 (1.3)	1.03	0.67
Insulin use, %	27.2%	22.8%	1.27	0.20
Obese (BMI > 30 kg/m^2^), %	77%	64.8%	1.82	< 0.01
Alcohol problem, %	12.1%	6.8%	1.88	0.02
Cigarette smoking, %	24.3%	15.1%	1.79	< 0.01
Estrogen use, %	4.9%	4.0%	1.24	0.56
Low annual income, %	75.7%	54.8%	2.57	< 0.01
Duration of diabetes, years	11.1 (10.6)	10.0 (10.3)	1.01	0.20
Number of comorbidities	2.21 (1.7)	1.3 (1.3)	1.45	< 0.01
Number of prescription medications	8.7 (4.3)	6.1 (3.5)	1.19	< 0.01

Each cell contains either % or mean (standard deviation).

**Table 3:** Multivariate logistic regression: obstructive lung disease vs. obesity adjusted for potential confounders (*N* = 903)

Characteristic	OR	***P***	95% CI
**Obese**	**1.58**	**0.03**	**1.05, 2.37**
Gender (Male)	0.51	< 0.01	0.35, 0.74
Low income	1.79	< 0.01	1.20, 2.67
Number of comorbidities	1.18	0.01	1.04, 1.33
Number of prescription medications	1.12	< 0.01	1.07, 1.18
Cigarette smoking	1.34	0.19	0.86, 2.07
Alcohol problem	1.51	0.17	0.84, 2.70

Next, potential confounding variables associated with obstructive lung disease with *P* < 0.15 were included in a logistic regression model using obstructive lung disease prevalence as the outcome. This adjusted model showed a significant association between obstructive lung disease and obesity (odds ratio (OR) = 1.58; 95% CI = 1.05, 2.37; *P* = 0.03) (Table 3).

To test the hypothesis that the association between obstructive lung disease and obesity is mainly observed in women, we ran a stratified analysis by gender. This adjusted multivariate logistic regression model showed a highly significant association between obstructive lung disease prevalence and obesity in women (OR = 2.18; 95% CI = 1.27, 3.72; *P* = 0.01) but not in men (OR = 0.97; 95% CI = 0.51, 1.83; *P* = 0.93) (Table 4).

**Table 4:** Multivariate logistic regression of dependent variable obstructive lung disease vs. obesity and the confounders, stratified by gender

Characteristic	Adjusted Odds Ratio	***P***	95% confidence interval
**Women (*N*** **= 490)**			
**Obese**	**2.18**	**0.01**	**1.27, 3.72**
Low income	1.44	0.16	0.87, 2.40
Number of comorbidities	1.24	0.01	1.05, 1.45
Number of prescription medications	1.12	0.00	1.05, 1.20
Cigarette smoking	1.60	0.10	0.91, 2.80
Alcohol problem	1.47	0.40	0.60, 3.57
**Men (*N*** **= 413)**			
**Obese**	**0.97**	**0.93**	**0.51, 1.83**
Low income	2.58	0.00	1.35, 4.92
Number of comorbidities	1.11	0.31	0.91, 1.34
Number of prescription medications	1.14	0.00	1.05, 1.23
Cigarette smoking	1.02	0.96	0.50, 2.10
Alcohol problem	1.71	0.18	0.79, 3.77

## Discussion

In this population of primary care patients with diabetes, obesity and obstructive lung disease were significantly and positively associated, with the effect being observed primarily in women. Obesity has been shown to be important in the association between asthma and type 2 diabetes, but the mechanism linking the three chronic conditions is poorly understood [33, 34]. Asthma can interact synergistically with obesity, increasing circulating levels of inflammatory cytokines and leading to an increased risk of insulin resistance and type 2 diabetes [35]. However, asthma has been associated with an increased risk of type 2 diabetes in women, regardless of BMI, suggesting that chronic inflammation may contribute to the pathogenesis of diabetes [36].

Other studies have shown that the asthma–obesity association is stronger in women. Some studies have suggested that estrogen may act directly on the immune system to increase the severity of asthma symptoms [37], or may act indirectly to damage lung mechanics and increase inflammation [38]. Endogenous estrogen is unlikely to be the cause of our findings since the majority of the women were post-menopausal, and exogenous estrogen use was not significantly associated with lung disease (Table 2). Anatomy may play a role in the female risk for obstructive lung disease. Women usually have smaller lungs than men. Hence, irritants like cigarette smoke or workplace dust, allergens, and fumes enter the lungs at a higher concentration [39].

This study has several strengths. First, the interviewed subjects were a randomly selected subset of patients receiving primary care in the northeast US, and they are therefore likely to be representative of primary care patients. Second, BMI was obtained by direct measurement of height and weight using standard techniques. This study does, however, also have several limitations, including a self-report of obstructive lung disease, lack of confirmation of the obstructive lung disease diagnoses, inability to distinguish between asthma and COPD, lack of information on the time relation between onset of obstructive lung disease and obesity, and a cohort mainly composed of white race. As in any cross-sectional study, unmeasured confounders could contribute to the apparent associations found.

## Conclusion

Our findings suggest a possible association between obstructive lung disease and obesity in diabetes, particularly among women. These results raise the possibility that obstructive lung disease, especially in women, could be controlled by interventions targeting obesity. More research is needed to identify the mechanisms at work.

## Data Availability

All data analyzed during this study are included in this published article.

## Abbreviations

VDIS: Vermont Diabetes Information System
BMI: Body mass index
COPD: Chronic obstructive pulmonary disease
A1C: Glycosylated hemoglobin
CI: Confidence interval
SD: Standard deviation
N: Number of subjects with the characteristic
OR: Odds ratio
